# Single Incision Laparoscopic Surgery for Acute Appendicitis: Feasibility in Pediatric Patients

**DOI:** 10.1155/2010/294958

**Published:** 2010-02-04

**Authors:** Andre Chow, Omer Aziz, Sanjay Purkayastha, Ara Darzi, Paraskevas Paraskeva

**Affiliations:** Division of Surgery, Imperial College London, St Mary's Hospital Campus, Praed Street, London W2 1NY, UK

## Abstract

*Background*. Laparoscopic appendicectomy is accepted by many as the gold standard approach for the treatment of acute appendicitis. The use of Single Incision Laparoscopic Surgery (SILS) has the potential of further reducing postoperative port site complications as well as improving cosmesis and patient satisfaction. *Method*. In this paper we report our experience and assess the feasibility of SILS appendicectomy in the pediatric setting. *Results*. Five pediatric patients with uncomplicated appendicitis underwent SILS appendicectomy. There were no significant intraoperative or postoperative complications. All patients were discharged within 24 hours. *Conclusions*. The use of Single Incision Laparoscopic Surgery appears to be a feasible and safe technique for the treatment of uncomplicated appendicitis in the pediatric setting. Further studies are warranted to fully investigate the potential advantages of this new technique.

## 1. Introduction

The rapid uptake of minimally invasive techniques has affected many areas of surgery, including the management of acute appendicitis. Laparoscopic appendicectomy is also a standard and recognised technique in the paediatric setting, with some surgeons advocating a primarily laparoscopic approach to all paediatric patients presenting with appendicitis [1]. Initial fears regarding the possibility of increased rates of postoperative complications seem to have been dispelled with improved instrumentation, technique, and growing experience both from the surgeon and ancillary staff [2]. In fact, although operating times and cost may be increased with the laparoscopic approach, this may be offset by a reduced postoperative stay compared to the standard open approach [3]. 

Single Incision Laparoscopic Surgery (SILS) is a new technique through which laparoscopic surgery takes place through a single umbilical incision, without the need for additional laparoscopic ports. This new method has been used for a variety of laparoscopic operations including tubal ligation [4], hysterectomy [5], appendicectomy [6, 7], cholecystectomy [8], sleeve gastrectomy [9], colectomy [10], and nephrectomy [11]. The single incision technique has the possible advantages of reduced postoperative pain, faster return to normal function, reduced port site complications, and improved cosmesis and patient satisfaction. 

In this paper we present our first experiences and assess the feasibility of using SILS to treat appendicitis in the pediatric population. 

## 2. Patients

SILS appendicectomy was carried out in 5 children in a teaching hospital in central London. All patients had a body mass index between 20 and 25, and all operations were carried out by the same consultant surgeon. 

The first patient was a 12-year-old boy, who presented with a single day history of central abdominal pain that localised to the right iliac fossa. On admission his white cell count and CRP were both within normal range, but he was tender with localised peritonism in the right iliac fossa. His symptoms did not improve overnight and thus the decision was made to proceed with laparoscopy.

The second patient was a 14-year-old girl who presented with a 5-day history of worsening right iliac fossa pain with localised peritonism. She had a normal white cell count, but a raised CRP of 29 on admission and was booked for laparoscopy

The third patient was a 13-year-old boy with a 2-day history of right iliac fossa pain. His white cell count and CRP were within the normal range. However, his symptoms worsened overnight and thus he was booked for laparoscopy. The fourth patient was a 12-year-old girl with a 1 day history of abdominal pain and normal white cell count and CRP. Her symptoms also worsened overnight, and thus we proceeded to laparoscopy. The fifth and final patient was a 13-year-old boy presenting with a 12-hour history of pain and raised white cell count of 15.

## 3. Technique

Access was gained via an open umbilical incision. Firstly the umbilicus was everted using a Littlewoods forcep. The incision was made either vertically or transversely, with a Prolene (Ethicon, New Brunswick, NJ, USA) stay suture placed either side of the incision. Care was taken to keep the incision within the umbilical ring for the best cosmetic outcome. A mixture of sharp and blunt dissection was used down to the linea alba which was incised. The peritoneum was opened under direct vision, and a 11 mm laparoscopic port inserted. Pneumoperitoneum was then established. A 5 mm 30 degree laparoscope was used to complete a full laparoscopy. Up to 2 further 5 mm DEXIDE (Covidien, Mansfield, Massachusetts, USA) ports were then inserted through the fascia to either side of the 11 mm port. 

Mobilisation of the appendix was achieved with the use of Roticulator instruments (Covidien, Mansfield, Massachusetts, USA). A window was made in the mesoappendix near the appendix base, and the appendix and mesoappendix both stapled and divided using an EndoGIA stapler (Covidien, Mansfield, Massachusetts, USA). In our third and fourth patients this procedure was assisted by the placement of a single suture, placed through the abdominal wall in the right iliac fossa. The needle was then passed through the mesoappendix near the appendix base, before being passed back up through the skin again. This formed a sling to retract the appendix ventrally, allowing easier positioning of the EndoGIA stapler (Covidien, Mansfield, Massachusetts, USA). All specimens were removed with the use of an EndoCATCH bag (Covidien, Mansfield, Massachusetts, USA). Irrigation was carried out as required. 

Closure of the wound was performed in layers, with absorbable sutures to both fascia and skin.

## 4. Results

SILS appendicectomy took an average of 56.4 minutes to perform (80, 48, 65, 50, and 45 minutes for patients 1, 2, 3, 4, and 5, resp.). The first patient had a macroscopically normal looking appendix. No other intra-abdominal pathology could be found and it was decided to proceed to appendicectomy. Following surgery, the patient symptoms improved and he was discharged on postoperative day 1. The other four patients all had macroscopically inflamed appendixes without evidence of gangrene or perforation. There were no significant intraoperative complications in any patients, and no need for conversion to standard laparoscopic appendicectomy. All patients were discharged within 23 hours and were brought back to clinic 6 to 8 weeks later for out-patient review. There were no postoperative wound infections, intra-abdominal abscess formation, or episodes of small bowel obstruction. Anecdotally all patients and their parents were very satisfied with their operative management, and particularly enthusiastic in regard to the single incision approach. On follow-up in clinic, the umbilical scar was very difficult to visualise once healing had been completed.

## 5. Discussion

Laparoscopic appendicectomy is now accepted as the gold standard for treatment of acute appendicitis in many centres. The laparoscopic approach has been demonstrated to have lower wound infection rates postoperatively, as well as having significant gains in terms of length of hospital stay and return to normal function [12]. Laparoscopic appendicectomy is also associated with a lower rate of adhesional bowel obstruction compared with the open approach [13]. Initial worries regarding rates of intra-abdominal abscess formation seem to have been refuted by recent studies [3], and it is the authors viewpoint that good peritoneal irrigation is actually aided by the improved intra-abdominal view offered with laparoscopy.

Single incision laparoscopic surgery (SILS) is a new technique that has now been utilised in many centres for appendicectomy. We have previously detailed our initial experiences with the use of SILS for appendicectomy and cholecystectomy in the adult population [14, 15]. The major difficulty with this new technique is the sacrifice that has to be made in terms of comfort and ergonomics. As all instruments and camera are inserted through the same incision, the ability to triangulate your instruments around the target is lost. Although this can be partially rectified by the use of roticulator instruments, the surgeon ends up working with his hands very close together, and finds himself often being impeded by the laparoscope and the assistant. Similarly, the surgeon's right hand will control the left-sided instrument on the screen and the left hand controls the right-sided instrument on screen. These technical difficulties do make SILS a more demanding procedure on the operating surgeon than normal laparoscopic techniques. In our experience this led to an initial significant increase in the operation time. However, with increasing exposure to the technique, operating times have been reduced significantly, and are now very similar to the average time taken for a standard laparoscopic appendicectomy. Future improvements in instrumentation may help to reduce operating times further. 

Although the small size, and limited age range of the patients in this series, precludes any meaningful statistical analysis, it does demonstrate that the SILS approach may be feasible for particular cohorts within the pediatric population. This supports the results of other groups using the SILS approach in pediatric patients [16, 17]. However, applicability of SILS to very young patients was not assessed in this paper. This series also adds further to the current literature demonstrating the applicability of SILS in uncomplicated appendicitis. In the future prospective studies with sufficient power are now warranted to demonstrate any statistically significant benefits over the standard laparoscopic method. These are most likely to be in terms of postoperative pain, port site complications, cosmesis, and patient satisfaction.

## References

[1] Sauerland S, Lefering R, Neugebauer EA (2004). Laparoscopic versus open surgery for suspected appendicitis. *Cochrane Database of Systematic Reviews*.

[2] Partrick DA (2006). Prospective evaluation of a primary laparoscopic approach for children presenting with simple or complicated appendicitis. *American Journal of Surgery*.

[3] Ikeda H, Ishimaru Y, Takayasu H, Okamura K, Kisaki Y, Fujino J (2004). Laparoscopic versus open appendectomy in children with uncomplicated and complicated appendicitis. *Journal of Pediatric Surgery*.

[4] Wheeless CR (1972). Outpatient laparoscope sterilization under local anesthesia. *Obstetrics and Gynecology*.

[5] Pelosi MA, Pelosi MA (1992). Laparoscopic supracervical hysterectomy using a single-umbilical puncture (mini-laparoscopy). *The Journal of Reproductive Medicine*.

[6] Esposito C (1998). One-trocar appendectomy in pediatric surgery. *Surgical Endoscopy*.

[7] Rispoli G, Armellino MF, Esposito C (2002). One-trocar appendectomy: sense and nonsense. *Surgical Endoscopy and Other Interventional Techniques*.

[8] Navarra G, Pozza E, Occhionorelli S, Carcoforo P, Donini I (1997). One-wound laparoscopic cholecystectomy. *British Journal of Surgery*.

[9] Reavis KM, Hinojosa MW, Smith BR, Nguyen NT (2008). Single-laparoscopic incision transabdominal surgery sleeve gastrectomy. *Obesity Surgery*.

[10] Bucher P, Pugin F, Morel P (2008). Single port access laparoscopic right hemicolectomy. *International Journal of Colorectal Disease*.

[11] Rane A, Rao P, Rao P (2008). Single-port-access nephrectomy and other laparoscopic urologic procedures using a novel laparoscopic port (R-port). *Urology*.

[12] Keus F, de Jong JA, Gooszen HG, van Laarhoven CJ (2006). Laparoscopic versus open cholecystectomy for patients with symptomatic cholecystolithiasis. *Cochrane Database of Systematic Reviews*.

[13] Tsao KJ, St Peter SD, Valusek PA (2007). Adhesive small bowel obstruction after appendectomy in children: comparison between the laparoscopic and open approach. *Journal of Pediatric Surgery*.

[14] Chow A, Purkayastha S, Aziz O, Paraskeva P Single-incision laparoscopic surgery for cholecystectomy: an evolving technique.

[15] Chow A, Purkayastha S, Paraskeva P (2009). Appendicectomy and cholecystectomy using single-incision laparoscopic surgery (SILS): the first UK experience. *Surgical Innovation*.

[16] Ponsky TA, Diluciano J, Chwals W, Parry R, Boulanger S (2009). Early experience with single-port laparoscopic surgery in children. *Journal of Laparoendoscopic and Advanced Surgical Techniques*.

[17] Visnjic S (2008). Transumbilical laparoscopically assisted appendectomy in children: high-tech low-budget surgery. *Surgical Endoscopy and other Interventional Techniques*.

